# A method of assessing the sensitivity of the Cochran–Mantel–Haenszel test to an unobserved confounder

**DOI:** 10.1098/rsta.2008.0030

**Published:** 2008-04-11

**Authors:** Binbing Yu, Joseph L. Gastwirth

**Affiliations:** 1 Laboratory of Epidemiology, Demography and Biometry,National Institute on AgingBethesda, MD 20892, USA; 2 Department of Statistics, The George Washington UniversityWashington, DC 20037, USA

**Keywords:** Cochran–Mantel–Haenszel test, confounders, sensitivity analysis, stratification

## Abstract

Observational studies, including the case-control design frequently used in epidemiology, are subject to a number of biases and possible confounding factors. Failure to adjust with them may lead to an erroneous conclusion about the existence of a causal relationship between exposure and disease. The Cochran–Mantel–Haenszel (CMH) test is widely used to measure the strength of the association between an exposure and disease or response, after stratifying on the observed covariates. Thus, observed confounders are accounted for in the analysis. In practice, there may be causal variables that are unknown or difficult to obtain. Hence, they are not incorporated into the analysis. Sensitivity analysis enables investigators to assess the robustness of the findings. A method for assessing the sensitivity of the CMH test to an omitted confounder is presented here. The technique is illustrated by re-examining two datasets: one concerns the effect of maternal hypertension as a risk factor for low birth weight infants and the other focuses on the risk of allopurinol on having a rash. The computer code performing the sensitivity analysis is provided in appendix A.

## 1. Introduction

Observational studies are commonly used to evaluate the effect of treatments or chemical exposure, e.g. the association between risk factors and a subsequent event. Unlike randomized experiments, researchers cannot control the assignment of treatments to subjects to ensure that subjects receiving different treatments or exposure are similar with respect to major covariates. The Cochran–Mantel–Haenszel (CMH; Cochran 1954; Mantel & Haenszel 1959) test is widely used to test the strength of the association after controlling for the observed confounders. The data are stratified into multiple 2×2 tables by known confounders, where in each stratum the distributions of the confounders in both groups are similar.

Since observational studies remain subject to unobserved confounding, one should explore how sensitive the results are to a possible omitted variable. Extensive work has been done on how to perform sensitivity analysis for observational data by Cornfield *et al*. (1959), Rosenbaum & Rubin (1983, 1984), Rosenbaum & Krieger (1990), Gastwirth (1992), Rosenbaum (2002) and others. Most of the literature models the association between an unobserved confounder, denoted by *U*, and treatment assignment, and then recomputes the test statistics and *p* values for a range of plausible differences in the prevalences of the confounder in the two groups. If a moderate degree of confounding could change the inference about the treatment effect on the outcome, then one questions the soundness of the conclusions. Gastwirth *et al*. (1998) proposed a dual and simultaneous sensitivity analysis for matched pair data, which extended a strengthened form of the original Cornfield inequality (Gastwirth 1988, p. 296). Both the relationship between *U* and treatment assignment and that between *U* and response are jointly modelled. This simultaneous form allows one to incorporate subject matter knowledge about both relationships into the sensitivity analysis.

This paper extends the ‘simultaneous’ approach to examine the sensitivity of the CMH test to an unobserved confounder. The rest of this paper is organized as follows. Section 2 reviews the CMH test and describes the stratified model for the simultaneous relationships. Then we apply the sensitivity analysis to a dataset examining maternal hypertension as a risk factor for low birth weight (LBW) infants and another dataset concerned with a side effect (rash) of allopurinol in §3. We discuss the potential use and future development of sensitivity analysis in observational studies in §4.

## 2. Sensitivity analysis for the CMH test

Let *X* be the exposure, e.g. environmental hazard, genetic factor, radiation exposure; and *Y* be the outcome, e.g. disease, mortality, or in an employment discrimination case, being promoted or passing a pre-employment exam. We are interested in testing the causal relationship between *X* and *Y* using the CMH test to control for other known factors related to the response. Usually the data are organized into *J* 2×2 tables, where in each stratum the covariates have similar values. The data for the *j*th stratum (*j*=1, …, *J*) are shown below[Table tbl6].

Let *π*_1*j*_ (*π*_0*j*_), *j*=1, …, *J*, be the probability that subjects who are exposed (not exposed) in stratum *j* has an event. In terms of these parameters, the odds ratio between exposure and outcome in stratum *j* is

$$\theta_{j}=\pi_{1j}(1-\pi_{0j})/[\pi_{0j}(1-\pi_{1j})],\;j=1,\ldots ,J.$$

Furthermore, assume that the odds ratio has a common value *θ*. Let $D=\{a_{j},b_{j},c_{j},d_{j},\;j=1,\ldots ,J\}$ be the observed data for the *J* 2×2 tables. When the responses for each group in all strata follow independent binomial distributions, the full data have the product binomial likelihood function

(2.1) $$L(\theta |D)=\prod_{j=1}^{J}\left(\begin{array}{c} m_{1j}\\ a_{j} \end{array}\right)\pi_{1j}^{a_{j}}{\left(1-\pi_{1j}\right)}^{b_{j}}\left(\begin{array}{c} m_{0j}\\ c_{j} \end{array}\right)\pi_{0j}^{c_{j}}{\left(1-\pi_{0j}\right)}^{d_{j}}.$$

From (2.1), we have $E(a_{j})=m_{1j}\pi_{1j}$, $V(a_{j})=m_{1j}\pi_{1j}(1-\pi_{1j})$, $E(c_{j})=m_{0j}\pi_{0j}$ and $V(c_{j})=m_{0j}\pi_{0j}(1-\pi_{0j})$.

If all the confounders are observed and controlled by stratification, then the null hypothesis of no-exposure effect is *H*_0_: *π*_0*j*_=*π*_1*j*_, *j*=1, …, *J* or *H*_0_: *θ*=1. Cochran's (1954) statistic for testing *H*_0_: *θ*=1 versus *H*_1_: *θ*>1 is

(2.2) $$W_{0}=\frac{\sum_{j=1}^{J}w_{j}(p_{1j}-p_{0j})}{{\left[\sum_{j=1}^{J}w_{j}{\bar{p}}_{j}(1-{\bar{p}}_{j})\right]}^{1/2}},$$

where *w*_*j*_=*m*_1*j*_*m*_0*j*_/*N*_*j*_, ${\bar{p}}_{j}=(m_{1j}p_{1j}+m_{0j}{\hat{p}}_{0j})/N_{j}$ and $p_{1j}=a_{j}/m_{1j}$, $p_{0j}=c_{j}/m_{0j}$ are the estimates of *π*_1*j*_ and *π*_0*j*_, respectively. Using the conditional central hypergeometric likelihood rather than the product binomial (2.1), Mantel & Haenszel (1959) proposed a similar test. The two tests are asymptotically equivalent and are often referred to as the CMH test. The CMH test is known to be the optimal method of combining multiple 2×2 tables with common odds ratio (Woolson *et al*. 1986).

The large sample distribution of (2.2) converges to a standard normal distribution under the null hypothesis that *θ*=1 (Cochran 1954). More generally, the statistic

(2.3) $$W=\frac{\sum_{j=1}^{J}w_{j}[(p_{1j}-p_{0j})-(\pi_{1j}-\pi_{0j})]}{{\left\{\sum_{j=1}^{J}w_{j}^{2}\left[\frac{\pi_{1j}(1-\pi_{1j})}{m_{1j}}+\frac{\pi_{0j}(1-\pi_{0j})}{m_{0j}}\right]\right\}}^{1/2}},$$

converges to a standard normal distribution as *N*_*j*_→∞ (Woolson *et al*. 1986).

Sometimes, information on known risk factors is not available or other risk factors related to the response have not been discovered. Here we assume that there is an unobserved factor *U* and that the relationship between binary outcome *Y* and exposure *X* and confounder *U* is modelled by

(2.4) $$\pi_{xj|u}=P(Y=1|X=x,S=j,U=u)=\frac{\text{exp}\;(\alpha_{j}+\beta x+\gamma u)}{1+\text{exp}\;(\alpha_{j}+\beta x+\gamma u)},$$

where *γ* is the effect of *U*, which is called the strength parameter (Yu & Gastwirth 2005), and *β* is the true effect of exposure *X*. The null hypothesis of no-exposure effect is *H*_0_: *β*=0 and the alternative hypothesis is *H*_1_: *β*≠0.

The unobserved variable *U* may also be associated with the exposure. Assuming that *U* is binary, the prevalence of *U* by exposure level in each stratum is modelled by

(2.5) $$P(U=1|X=x,S=j)=\frac{\text{exp}\;(\lambda_{j}+\delta_{j}x)}{1+\text{exp}\;(\lambda_{j}+\delta_{j}x)}.$$

Here, the *δ*_*j*_, indicating the association between *X* and *U* in each stratum, are called the imbalance parameters (Yu & Gastwirth 2005). To simplify the calculation, we assume that the imbalance parameters *δ*_*j*_ are equal to *δ*. Then, $q_{j}={\text{e}}^{\lambda_{j}}/(1+{\text{e}}^{\lambda_{j}})$ is the prevalence of *U* in the non-exposed group in stratum *j*. When the confounder *U* is omitted in the analysis, the marginal probability of response at each exposure level *x* in stratum *j* becomes

$$\pi_{xj}=\frac{\text{exp}\;(\alpha_{j}+\beta x+\gamma )}{1+\text{exp}\;(\alpha_{j}+\beta x+\gamma )}\frac{\text{exp}\;(\lambda_{j}+\delta x)}{1+\text{exp}\;(\lambda_{j}+\delta x)}+\frac{\text{exp}\;(\alpha_{j}+\beta x)}{1+\text{exp}\;(\alpha_{j}+\beta x)}\frac{1}{1+\text{exp}\;(\lambda_{j}+\delta x)}.$$

The true null hypothesis of no-exposure effect is *H*_0_: *β*=0, which is really the hypothesis of interest. Note that *H*_0_: *π*_0*j*_=*π*_1*j*_, *j*=1, …, *J*, and *H*_0_: *β*=0 are not equivalent when there is an omitted confounder. If *U* is not a confounder, i.e. *δ*=0 or *γ*=0, then the equalities *π*_1*j*_=*π*_0*j*_, *j*=1, …, *J*, still hold under the null hypothesis *H*_0_: *β*=0, so that the CMH test *C*_0_ is indeed testing the effect of exposure. When *U* is indeed a confounder, it has been shown that, if both *γ*>0 and *δ*>0, then *π*_1*j*_=*π*_0*j*_, *j*=1, …, *J*, even when the true null hypothesis holds (Yu & Gastwirth 2005) and the CMH test would be biased. This implies that confounder *U* could induce spurious positive association between exposure and outcome. However, if *γ*>0 and *δ*<0 then the true association between *X* and *Y* will be underestimated if the confounder is omitted.

If the strength parameter *γ*, the imbalance parameter *δ* and the prevalences *q*_1_, …, *q*_*J*_ of the confounder are known or can be reliably estimated from other sources, the estimates of *π*_1*j*_ and *π*_0*j*_ can be calculated from the likelihood (2.1) under the null hypothesis *H*_0_: *β*=0 (see appendix A). Then the correct CMH statistic is obtained by substituting the estimates $\left({\hat{\pi }}_{1j},{\hat{\pi }}_{0j}\right)$ for $\left(\pi_{0j},\pi_{1j}\right)$ in (2.3) as the effect of *U* is now incorporated into the response probabilities *π*_*xj*_. In practice these quantities are unknown, so one assumes a possible range for (*q*_1_, …, *q*_*J*_, *γ*, *δ*); the test statistic and the *p* value are calculated using (2.3). A table showing the *p* values with respect to different parameters for confounding can be used to assess the plausibility that the current inference could be altered due to the effect of an unobserved factor.

## 3. Application

The proposed sensitivity analysis will be applied to data from two observational studies. In these applications, *C*=exp (*γ*) measures the effect of *U* on binary response *Y* and *D*=exp (*δ*) measures the increasing odds of *U* being positive when *X* increases by one unit.

### (a) Sensitivity analysis of maternal hypertension as a risk factor for LBW infants

Data on 500 singleton births in a London Hospital (Hills & De Stavola 2002) were used to examine the sensitivity of the association of maternal hypertension as a risk factor for LBW infants. The dataset provides the birth information of 500 singletons with the following eight variables: identity number for mother and baby, birth weight of baby (indicator for birth weight less than 2500 g), gestation period (indicator for gestation period less than 37 weeks), maternal age, indicator for maternal hypertension and sex of baby (1, male; and 2, female). Ten mothers with missing gestation period information were dropped from the analysis. The exposure of interest (*X*) is maternal hypertension and the event of interest is LBW infant (*Y*).

‘Advanced’ maternal age is defined as any expectant mother who will have reached her 35th birthday by delivery date. The mothers are divided into ‘young’ and ‘advanced’ age group according to this criterion. The number of infants with normal birth weight (NBW) and LBW by sex, gestation status, maternal age and maternal hypertension are shown in table 1. The CMH test is significant with *p*=0.015 and the odds ratio is 2.74 with 95% CI (1.23, 6.14). The Breslow–Day test for homogeneity shows that the odds ratio estimates are homogeneous across different strata (*p*=0.785). Is it safe to draw the conclusion that maternal hypertension is causally related to LBW?

Despite substantial reductions in US infant mortality during the past several decades, black–white disparities in infant mortality rates persist. Important determinants of racial/ethnic differences in infant mortality are LBW, defined as less than 2500 g (US Department of Health and Human Services 2000). The number of LBW infants among 1000 live births for the whites are 5.7–6.5 and for the blacks are 12.7–13.0 from 1980 to 2000 (Centers for Disease Control and Prevention 2002; http://www.cdc.gov/mmwr/preview/mmwrhtml/mm5127a1.htm). Hence, race is an important risk factor with an apparent odds ratio of approximately 2.0–2.4 for LBW (David & Collins 1997). A recent study (Geronimus *et al*. 2007) also showed that being black increases the odds ratio of hypertension by 2.11–4.04 for women between 15 and 65 years of age. Also, LBW is related to many other risk factors, e.g. the use of assisted reproductive technology, use of alcohol or drugs. Here, we focus on the race as the unobserved confounder (*U*) because it is a recognized risk factor. We assume that the mother's race is not related to the sex of their babies, but that the percentage of mothers who are black varies across the maternal age groups as well as gestation term. The possible scenarios for the percentage of black mothers by maternal age and gestation term are shown in table 2. In scenarios 1 and 2, we assume that mothers with preterm babies consist of a higher percentage of black women. In scenarios 3 and 4, we assume that there are more black mothers with normal maternal age. In scenario 5, the black mothers tend to be younger and have more preterm babies than whites.

Table 3 shows the two-sided *p* values of the adjusted CMH test with respect to different values of *C* and *D* for scenarios 1–5. Note that the strength parameter *C*=exp (*γ*) is the odds ratio of a black mother having a LBW baby compared with a white mother. The imbalance parameter *D*=exp (*γ*) is the increased odds ratio of a mother being black if she has hypertension. When *C*=1 or *D*=1, race is not a confounder and the CMH test is unchanged.

**Figure 1:**
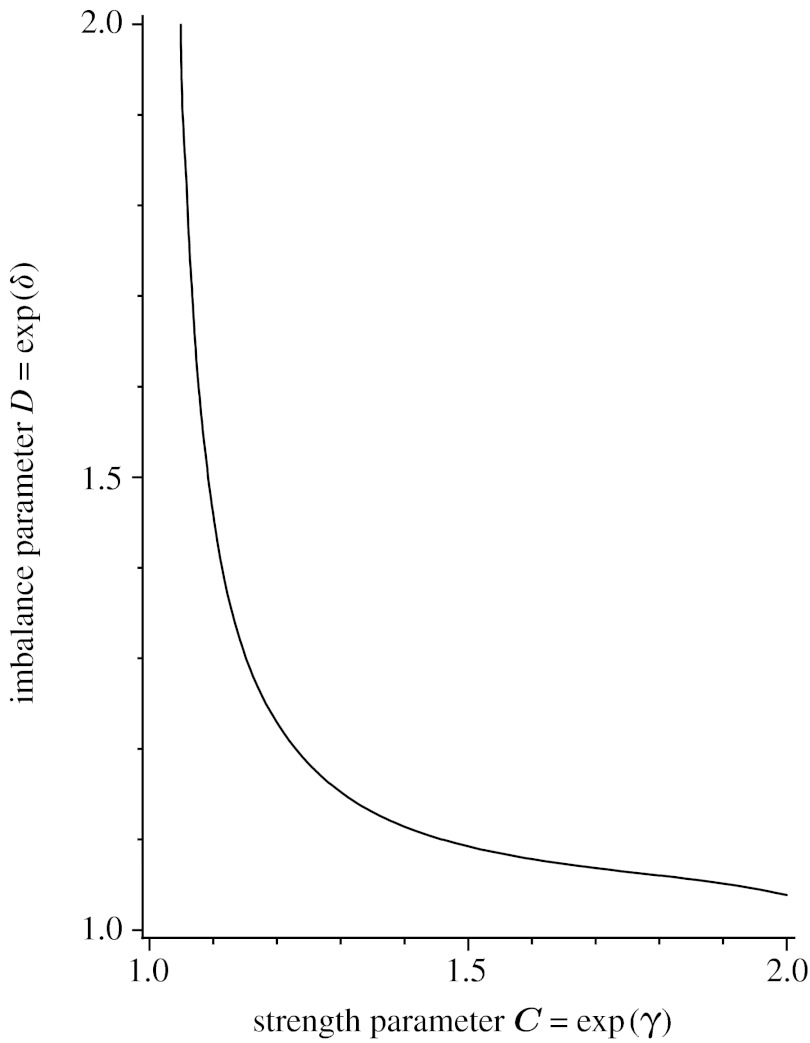
A plot of the values of the sensitivity parameters *C* and *D* required to raise the two-sided *p* value of the CMH test to 0.05.

Another way to perform the sensitivity analysis is to obtain the threshold values of the sensitivity parameters *C* and *D*, which increase the *p* value of the CMH test to 0.05. Figure 1 shows the plots of sensitivity parameters *C* and *D*, which corresponds to *p*=0.05. From figure 1, we see that when *C*=1.5 and *D*=1.1, then the two-sided *p* value would reach the 0.05 level. Thus, both table 3 and figure 1 show that the *p* value of the CMH test is sensitive to moderate changes in *C* and *D*, indicating that the significant association between maternal hypertension and LBW might have arisen from a hidden bias due to confounding. Hence, other studies, including race and ethnicity information, are needed to reach the conclusion that maternal hypertension causes LBW. If the studies demonstrate that increased incidence of black mothers with hypertension was slight with odds ratio less than 1.5, then the significance of the London study would not be affected by the omission of race.

Although in Rubin's causal model, sex and race are not used as ‘treatment’ variables in causal inference because it is difficult to ascribe causality to variables that cannot be altered (Holland 1986), here race is used as a proxy for other factors reflecting socio-economic disparities. More recently, race and sex have been used in the propensity score method to form ‘similar strata’ (Zanutto *et al*. 2005) but should not be the main potential outcome of a study as they cannot be altered.

### (b) Sensitivity analysis of allopurinol as a cause of rash

Rosenbaum (2002) examined the sensitivity of a study indicating that allopurinol can cause rash (Boston Collaborative Drug Project 1972). Allopurinol is commonly used for the treatment and prevention of attacks of gout and certain types of kidney stones. It is also used to treat elevated uric acid levels in the blood and urine, which can occur in patients receiving chemotherapy for the treatment of leukaemia, lymphoma and other types of cancer. Allopurinol is usually well tolerated by most patients; however, some may experience possible side effects of skin rash, hives and itching.

Table 4 shows the joint frequencies of drug use and getting a rash case for males and females, respectively. The CMH test is highly significant with *p*<0.0001. In a randomized trial or in the absence of unobserved confounders, this indicates strong evidence that allopurinol causes rash (Rosenbaum 2002, p. 131). Because the patients could also be taking amoxicillin and ampicillin, which are known to cause rash, is it possible that the rash was caused by an unobserved factor, e.g. use of amoxicillin or other drugs, instead of allopurinol?

Although information about the other drug use and users' allergic history was not collected, one can assess the sensitivity of the CMH test with respect to an unobserved confounder. Let *U* be the binary indicator for amoxicillin use, e.g. use amoxicillin (1) and no amoxicillin (0). Again, the prevalences of using amoxicillin among the non-allopurinol users are denoted by *q*_*j*_, *j*=1, …, *J*. The parameter *δ* indicates the imbalance of *U* between allopurinol users and non-users. The parameter *γ* indicates the increasing probability of having rash among users of amoxicillin. We assume two possible scenarios for the prevalences of amoxicillin use among the non-allopurinol users, i.e. *q*_*j*_=10% or 20%, where *j*=1, 2.

The increasing odds ratio of having rash for amoxicillin users compared with non-users is not known, but it is likely to be below 4. Based on table 5, we can see that the CMH test of allopurinol causing rash is not sensitive to the omitted variable (use of amoxicillin). Even when the strength parameter *C*=4 and the imbalance parameter *D*=4, the CMH test remains highly significant with *p*=0.002. This example also illustrates the additional information provided by the simultaneous sensitivity analysis compared with the earlier approaches by Cornfield *et al*. (1959) and Rosenbaum (2002), which focus primarily on the imbalance parameter and assume a confounder with infinitely large strength. Rosenbaum (2002, p. 131) used the parameter *Γ*, which is similar to *D* in our analysis, to measure the imbalance of treatment assignment with respect to the unobserved factor. The upper bounds on the *p* values based on his analysis are 0.036 and 0.30, for *Γ*=2 and 3, respectively, which indicates that the causal relationship between allopurinol use and rash could have arisen from an unobserved confounder that triples the risk of rash. While such a confounding is unlikely to exist, the conclusion based on the simultaneous analysis is stronger. Even when *C*=∞, the test barely reached non-significance for *D*=3 and *q*_*j*_=10%. This sensitivity analysis is more stringent, indicating the conclusion that allopurinol causes rash is very robust to an unobserved confounder. This is consistent with the current medical practice that lists rash as the one common side effect of allopurinol use (http://www.nlm.nih.gov/medlineplus/druginfo/medmaster/a682673.html).

## 4. Discussion

Because the CMH test is widely used to analyse the data obtained in epidemiological studies, legal cases and social sciences, it is important to conduct a sensitivity analysis to answer whether that inference could *plausibly* be explained by an unobserved confounder before drawing a firm conclusion.

The proposed simultaneous sensitivity analysis approach is similar to the primal sensitivity analysis method by Rosenbaum (2002). The major difference is the introduction of the strength parameter. The primal sensitivity analyses closely parallel the theory of randomized experiments and have been developed for many statistical tests and estimators, e.g. the McNemar and Wilcoxon signed-rank tests and the Hodges–Lehmann estimator (Rosenbaum 2002). The simultaneous approach allows one to use more subject matter knowledge. However, it also requires more assumptions about both relationships. The simultaneous approach is especially useful when an observational study is challenged owing to failure to control for a particular unobserved variable, when there are plausible ranges for the values of *γ* and *δ* (Gastwirth *et al*. 1998).

At the design stage, it is also useful to examine the size and power of the corrected CMH test in the presence of omitted confounders in a range of realistic values of the strength and imbalance parameters. The calculation of size and power for the Cochran–Armitage trend test has been derived by Yu & Gastwirth (2005). A similar technique can be used for the CMH test with omitted variable.

In future studies, we plan to extend this methodology of sensitivity analysis to data where the unobserved confounders are continuous and ordinal. In real-world observational studies, there may be more than one omitted confounder; background information on the joint distribution of the multiple confounders can then be used to replace the univariate distribution (2.5) in a sensitivity analysis.

## Figures and Tables

**Table 1:** Infants' birth weight by sex, gestation status, maternal age and hypertension.

			normal blood pressure		hypertension	
				
sex	gestation	maternal age	NBW	LBW	NBW	LBW
male	termed	young	98	2	15	2
		advanced	87	6	13	2
	preterm	young	7	5	2	4
		advanced	7	2	1	3
female	termed	young	91	5	13	1
		advanced	83	3	6	0
	preterm	young	4	6	2	5
		advanced	2	11	0	2

**Table 2:** The prevalences of black mothers by maternal age and gestation term for five possible scenarios.

			scenario (%)				
			
gestation	maternal age	prevalence	1	2	3	4	5
termed	normal	*q*_1_	10	5	10	20	10
	advanced	*q*_2_	10	5	5	10	5
preterm	normal	*q*_3_	20	10	10	20	20
	advanced	*q*_4_	20	10	5	10	10

**Table 3:** The effect of *U* on the *p* value of the CMH test for different values of *C* and *D*.

		*D*=exp (*δ*)			
		
scenario	*C*=exp (*γ*)	2	3	4	5
1	2	0.064	0.079	0.094	0.107
	3	0.078	0.110	0.140	0.170
	4	0.091	0.138	0.186	0.230
	5	0.103	0.165	0.227	0.287
2	2	0.057	0.066	0.074	0.083
	3	0.065	0.083	0.101	0.119
	4	0.072	0.099	0.127	0.156
	5	0.079	0.115	0.153	0.192
3	2	0.060	0.070	0.081	0.091
	3	0.070	0.092	0.114	0.136
	4	0.079	0.112	0.147	0.181
	5	0.087	0.131	0.177	0.223
4	2	0.067	0.085	0.100	0.114
	3	0.084	0.119	0.152	0.182
	4	0.098	0.150	0.199	0.244
	5	0.111	0.178	0.242	0.300
5	2	0.060	0.070	0.081	0.091
	3	0.070	0.092	0.114	0.136
	4	0.079	0.112	0.147	0.181
	5	0.087	0.131	0.177	0.223

**Table 4:** Drug use and rash cases by male and female.

sex	drug	no rash	rash
males	allopurinol	33	5
	other drugs	645	36
females	allopurinol	19	10
	other drugs	518	58

**Table 5:** One-sided *p* values of the CMH test for different values of (*γ*, *δ*).

*p* value	(*q*_1_, *q*_2_, *q*_3_, *q*_4_)							
	
	(10%, 10%, 10%, 10%)				(20%, 20%, 20%, 20%)			
		
	*C*=exp (*γ*)				*C*=exp (*γ*)			
		
*D*=exp (*δ*)	2	3	4	∞	2	3	4	∞
2	<0.001	<0.001	<0.001	0.005	<0.001	<0.001	<0.001	0.004
3	<0.001	<0.001	<0.001	0.057	<0.001	<0.001	<0.001	0.033
4	<0.001	<0.001	0.001	0.176	<0.001	<0.001	0.002	0.094
∞	0.004	0.046	0.161	0.999	0.005	0.045	0.130	0.890

**Table tbl6:** 

	outcome *Y*		
		
exposure group *X*	event (1)	non-event (0)	total
exposed (1)	*a*_*j*_	*b*_*j*_	*m*_1*j*_
not exposed (0)	*c*_*j*_	*d*_*j*_	*m*_0*j*_
total	*n*_1*j*_	*n*_0*j*_	*N*_*j*_

## Appendix A. Estimation of (*π*_1*j*_,*π*_0*j*_)

This appendix describes the estimation of (*π*_1*j*_,π_0*j*_) under the product-binomial likelihood (2.1) with fixed values of (*q*_1_, …, *q*_*J*_, *β*, *γ*, *δ*) for the allopurinol data in §3*b*. For stratum *j*, the prevalence of *U* in the non-exposed group is *q*_*j*_=exp (*λ*_*j*_)/[1+exp (*λ*_*j*_)]. Then the prevalence of *U* in the exposed group is $r_{j}=q_{j}{\text{e}}^{\delta }/(1-q_{j}+q_{j}{\text{e}}^{\delta })$. Let $A_{j}=\text{exp}\;(\alpha_{j}),\;B=\text{exp}\;(\beta ),\;C=\text{exp}\;(\gamma )$. Then for fixed values of (*β*, *δ*, *γ*, *q*_*j*_), the marginal probabilities of response are

(A1) $$\pi_{1j}=\frac{A_{j}BC}{1+A_{j}BC}r_{j}+\frac{A_{j}B}{1+A_{j}B}(1-r_{j})$$

and

(A2) $$\pi_{0j}=\frac{A_{j}C}{1+A_{j}C}q_{j}+\frac{A_{j}}{1+A_{j}}(1-q_{j}).$$

The log-likelihood of function (2.1) is $\text{log}\;L(\theta |D)=\sum_{j=1}^{J}\ell_{s}(\theta |D)$, where

$$\ell_{j}(\theta |D)=a_{j}\;\text{log}\;\pi_{1j}+b_{j}\;\text{log}\;(1-\pi_{1j})+c_{j}\;\text{log}\;\pi_{0j}+d_{j}\;\text{log}\;(1-\pi_{0j}),$$

for stratum *s*. The maximum-likelihood estimate (MLE) of *α*_*j*_, and $\left(\pi_{1j},\pi_{0j}\right)$ could be obtained from SAS PROC NLP. The code for the following 2×2×2 table from the allopurinol study with *q*_1_=*q*_2_=0.2, *β*=0, *γ*=log (2) and *δ*=log (2) is shown below. The MLEs of $\left(\pi_{1j},\pi_{0j}\right)$ are given in PIEST.

## References

[bib1] Boston Collaborative Drug Project (1972). Excess of ampicillin rashes associated with allopurinol or hyperuricemia. N. Engl. J. Med.

[bib2] Centers for Disease Control and Prevention (2002). Infant mortality and low birth weight among black and white infants—United States, 1980–2000. Morb. Mortal. Wkly Rep.

[bib3] Cochran W.G. (1954). Some methods for strengthening the common X2 tests. Biometrics.

[bib4] Cornfield J., Haenszel W., Lilienfeld A., Shimkin M., Wynder E. (1959). Smoking and lung cancer. J. Natl Cancer Inst.

[bib5] David R.J., Collins J.W. (1997). Differing birth weight among infants of U.S. born blacks, African-born blacks, and U.S. born whites. N. Engl. J. Med.

[bib6] Gastwirth J.L. (1988). Statistical reasoning in law and public policy.

[bib7] Gastwirth J.L. (1992). Methods for assessing the sensitivity of statistical comparisons used in Title VII cases to omitted variables. Jurimetrics.

[bib8] Gastwirth J.L., Krieger A.M., Rosenbaum P.R. (1998). Dual and simultaneous sensitivity analysis for matched pairs. Biometrika.

[bib9] Geronimus A.T., Keene D., Hicken M., Bound J. (2007). Black–white differences in age trajectories of hypertension prevalence among adult women and men, 1999–2002. Ethn. Dis.

[bib10] Hills M., De Stavola B. (2002). A short introduction to Stata 8 for biostatistics. http://www.timberlake.co.uk.

[bib11] Holland P.W. (1986). Statistics and causal inference. J. Am. Stat. Assoc.

[bib12] Mantel N., Haenszel W. (1959). Statistical aspects of retrospective studies of disease. J. Natl Cancer Inst.

[bib13] Rosenbaum P.R. (2002). Observational studies.

[bib14] Rosenbaum P.R., Krieger A. (1990). Sensitivity analysis for two-sample permutation inference in observational studies. J. Am. Stat. Assoc.

[bib15] Rosenbaum P.R., Rubin D.B. (1983). Assessing sensitivity to an unobserved binary covariate in an observational study with binary outcomes. J. R. Stat. Soc. Ser. B.

[bib16] Rosenbaum P.R., Rubin D.B. (1984). Reducing bias in observational studies using subclassification on the propensity score. J. Am. Stat. Assoc.

[bib18] US Department of Health and Human Services, 2000 *Healthy people 2010*, vols. 2 (conference ed.). Washington, DC: US Department of Health and Human Services.

[bib19] Woolson R.F., Bean J.A., Rojas P.B. (1986). Sample size for case-control studies using Cochran's statistic. Biometrics.

[bib20] Yu B., Gastwirth J.L. (2005). Sensitivity analysis for trend tests: application to the risk of radiation exposure. Biostatistics.

[bib21] Zanutto E.L., Lu B., Hornik R. (2005). Using propensity score subclassification for multiple treatment doses to evaluate a national anti-drug media campaign. J. Educ. Behav. Stat.

